# *Atractylodes lancea* rhizome derived exosome-like nanoparticles prevent alpha-melanocyte stimulating hormone-induced melanogenesis in B16-F10 melanoma cells

**DOI:** 10.1016/j.bbrep.2023.101530

**Published:** 2023-08-17

**Authors:** Tomoaki Ishida, Shumpei Morisawa, Kohei Jobu, Kei Kawada, Saburo Yoshioka, Mitsuhiko Miyamura

**Affiliations:** a Department of Pharmacy, Kochi Medical School Hospital, 185-1 Kohasu, Oko, Nankoku, Kochi, Japan; b Graduate School of Integrated Arts and Sciences, Kochi University, 185-1 Kohasu, Oko, Nankoku, Kochi, Japan

**Keywords:** B16-F10 cells, Melanogenesis, *Atractylodes lancea*, Exosome-like nanoparticles, miRNA

## Abstract

Aberrant melanin overproduction can significantly impact an individual's appearance and cause mental and psychological distress. Current inhibitors of melanin production exert harmful side effects due to inadequate selectivity; thus a need to develop more selective melanin synthesis inhibitors is necessary. Extracellular vesicles are important agents of intercellular signalling in prokaryotes and eukaryotes. Recently, plant-derived nanoparticles, similar to mammalian exosomes, have attracted attention for their use in health research. In this study, to investigate the potential of plant-derived exosome-like nanoparticles (ELNs) as inhibitors of melanin production, we used hot water to extract ELNs from the rhizome of *Atractylodes lancea* (A-ELNs). The size of A-ENLs ranged from 34 to 401 nm and carried three microRNA: ath-miR166f, ath-miR162a-5p, and ath-miR162b-5p. These A-ENLs were applied to B16-F10 melanoma cells treated with α-melanocyte-stimulating hormone (α-MSH). After A-ELNs were taken up by B16-F10 cells, their melanin levels were significantly reduced. Furthermore, A-ELNs significantly reduced tyrosinase activity in B16-F10 cells and mRNA expression of microphthalmia-associated transcription factor (*Mitf*), tyrosinase, tyrosinase-related protein 1, and DOPA chrome tautomerase. These results suggest that A-ELN suppresses melanogenic enzymes expression by downregulating *Mitf*, thereby inhibiting melanin synthesis. Hence, A-ELN can be developed into a novel topical drug after additional studies and optimization.

## Highlights

• Exosome like nanoparticles isolated from a hot water extract of *Atractylodes lancea* rhizomes (A-ELNs).
• A-ELNs contained three mature miRNAs (ath-miR166f, ath-miR162a-5p, ath-miR162b-5p).
• A-ELNs inhibited melanin production in α-MSH-stimulated B16-F10 cells.
• A-ELNs may have the potential as a new whitening agent in pharmaceuticals and cosmetics.

## 1 Introduction

Melanin is an important pigment that imparts colour to skin, eyes, and hair and protects cells from ultraviolet (UV) radiation. However, UV-B stimulation and paracrine cytokines, such as alpha-melanocyte stimulating hormone (α-MSH) and adrenocorticotropic hormone, induce excessive melanin synthesis. This leads to abnormal pigmentation and cosmetic concerns [[1], [2], [3]]. Suppressing melanin production can prevent such abnormalities and have a whitening effect. Melanin expression is induced by α-MSH secreted by keratinocytes exposed to UV-B and synthesized by melanocytes [4,5]. α-MSH affects melanocortin-1 receptors to activate cAMP signalling, inducing enzymes related to melanin synthesis to increase melanin levels [6].

In contrast, some ingredients, such as kojic acid and arbutin, block melanin production by inhibiting or modulating the activity of relevant enzymes; accordingly, both these ingredients are used in cosmetics and pharmaceuticals [7]. However, these enzyme inhibitors can produce several adverse effects, such as erythema, itching, and leukoderma [8,9]. Thus, a need to discover and develop new melanogenesis inhibitors without these side-effects is necessary.

Exosome-like nanoparticles (ELNs) are secreted by most types of animals, plants, and protist cells to transmit information between cells [10,11]. ELNs can also induce effects between different species, such as plant derived ELNs evoke physiological responses when administered to animals [12]. These ENLs may be involved in the physiological functions of some foods and medicinal plants. Furthermore, therapeutic effects of ELNs were reported in animal experiments [13].

Hence, the purpose of this study was to investigate the potential of using ELNs as inhibitors of melanin production in cosmetics and pharmaceuticals. Thus, we investigated the inhibitory effects of several plant-derived ELNs on melanin production in B16-F10 cells. Among them, we established that *Atractylodes lancea* rhizome-derived ELNs (A-ELNs) have inhibitory effects on melanin production. *A. lancea* is a perennial herb distributed throughout East Asia. Its rhizome has traditionally been used to treat nausea and digestive disorders, such as gastroparesis and gastric atony [14]. Furthermore, it exerts anti-inflammatory [15] and antitumoral effects [16]. However, none of the studies conducted have reported A-ELNs or evaluation of their medicinal effects. Thus, we investigated the inhibitory effect and mechanism of A-ELNs on α-MSH-induced melanogenesis in B16-F10 melanoma cells. Furthermore, in investigating the characteristics of A-ELNs, we attempted to identify the miRNAs contained in A-ELNs.

## 2 Materials and methods

### 2.1 Isolation and purification of *A-ELNs*

Dried rhizomes of *A. lancea* were purchased from Tsumura & Co. (Lot R15681, Tokyo, Japan). Twenty grams rhizome was added to 400 mL purified water and boiled for 30 min to obtain a hot water extract. The extract was centrifuged at 8000×*g* for 5 min, and the supernatant was collected and centrifuged at 15,000×*g* for 20 min. The final supernatant was filtered through a 0.8 μm filter (MilliporeSigma, Tokyo, Japan) and A-ELNs were extracted from the filtrate by exoEasy Maxi Kit (Qiagen, Hilden, Germany) and frozen at −70 °C.

### 2.2 Transmission electron microscopy (*TEM*) analysis

A-ENLs were examined using a JEM-2000EX operated at 100 kV (Japan Electron Optics Laboratory, Tokyo, Japan) at the Hanaichi UltraStructure Research Institute (Aichi, Japan). The Nanoparticle Characterization System (NanoSight, Malvern Instruments, UK) was used to determine the size distribution of extracellular vesicles (EVs).

### 2.3 *RNA* sequencing analysis

Total RNA was extracted from A-ELNs using the miRNeasy Mini Kit (Qiagen) according to the manufacturer's protocol. RNA sequencing was performed by Macrogen (Tokyo, Japan). The TruSeq Small RNA Library Prep kit (Illumina, San Diego, CA, USA) was used for library preparation following the manufacturer's instructions. The quality of RNA samples was tested on an Agilent 2100 Bioanalyzer (Agilent Technologies, Santa Clara, CA, USA). Libraries were sequenced on a HiSeq 2500 system (Illumina, San Diego, CA, USA). The sequence reads were filtered by removing low-quality reads, repeat sequences, and adaptor sequences to generate clean data. The reads were aligned to miRBase v22.1 (March 2022; http://www.mirbase.org) and RNAcentral v14.0 (March 2022; https://rnacentral.org) to classify known miRNAs and other types of RNA by comparing with the miRNA library of *Arabidopsis thaliana*. Reads with a randfold p-value of ≤0.05 for potential miRNA hairpin were used in further analysis [17]. Data are presented as the number of reads for each mature miRNA.

### 2.4 *B16-F10* melanoma cell culture

The immortalized mouse melanoma cell line B16-F10 was obtained from the American Type Culture Collection (ATCC, Manassas, VA, USA). B16-F10 cells were maintained at 37 °C, and 5% CO_2_ in Dulbecco's modified Eagle medium (Wako, Osaka, Japan) supplemented with 10% heat-inactivated, endotoxin-free fetal bovine serum (FBS), 100 U/mL penicillin, and 0.1 mg/mL streptomycin. Medium depleted of exosomes was obtained via ultracentrifugation at 110,000×*g* overnight at 4 °C and used in the experiment using A-ELNs.

### 2.5 Cell viability assay

To evaluate the cytotoxic effect of A-ELNs, B16-F10 cells (5.0 × 10^3^ cells/well) were plated in a 96-well microplate. After 24 h, B16-F10 cells were treated with different concentrations of A-ELNs (2.5, 5, 10, 20, or 40 μg/mL). After 48 h incubation, media containing A-ELNs were removed and 100 μL media containing 3-(4, 5-dimethylthiazol-2-yl)-2, 5-diphenyltetrazolium bromide (0.5 mg/mL) was added to the cells and incubated for another 1 h. Subsequently, the medium was removed, 100 μL dimethyl sulfoxide (DMSO) was added, and the absorbance was measured at 570 nm.

### 2.6 Confocal laser fluorescence microscopy

The A-ENL suspension was stained using the ExoSparkler Exosome Membrane Labelling Kit (Dojindo Laboratories, Kumamoto, Japan) according to the manufacturer's protocol. The labelled A-ELNs were added to B16-F10 cells plated in a glass dish (20 μg/mL) and incubated for 3 h. Nuclei were stained using 4′,6-Diamidino-2-phenylindole as a contrast stain and the cells were observed under a confocal laser scanning microscope (FV1000D IX81, Olympus, Tokyo, Japan).

### 2.7 Melanin synthesis inhibition

B16-F10 cells were cultured in 24-well plates (1.0 × 10^4^ cells/well). The cells were then treated with A-ELNs (5, 10, or 20 μg/mL) or the positive control, arbutin (1.0 mM) (Wako, Osaka, Japan), and simultaneously stimulated with 0.20 μM α-MSH, followed by incubation at 37 °C. After 72 h, the cells were washed twice with phosphate buffer and 1.0 M NaOH aqueous solution (0.30 mL) containing 10% DMSO was added to each well. The samples were incubated at 80 °C for 2 h, after which, absorbance at 360 nm was determined using a microplate reader. The melanin percentage in each sample was calculated as the ratio of absorbance compared to the untreated group.

### 2.8 Tyrosinase activity assay

B16-F10 cells were cultured in 24-well plates (1.0 × 10^4^ cells/well). The cells were then treated with A-ELNs (5, 10, or 20 μg/mL) or the positive control, arbutin (1.0 mM) (Wako, Osaka, Japan), and simultaneously stimulated with 0.20 μM α-MSH, followed by incubation at 37 °C. After 48 h, the cells were centrifuged to obtain cell pellets and resuspended in 1.0% Triton X-100 (Wako) aqueous solution (0.30 mL). The cell suspension was incubated 4 °C for 2 h. Next, samples were centrifuged at 13,000×*g* for 10 min at 4 °C to collect the supernatant. The supernatant was diluted by 1.0% Triton X-100 aqueous solution to 0.40 mg/mL after determining the protein concentration using the BCA Protein Assay Kit (Thermo Scientific, IL, USA). Next, 0.10 mL supernatant was mixed, in 1:1 ratio, with 0.10 mL of 10% (w/v) l-DOPA (Sigma Aldrich, Tokyo, Japan) in phosphate buffer and incubated at 37 °C. Subsequently, the absorbance was measured at 475 nm using a microplate reader. The percentage of cellular tyrosinase activity for each sample was calculated as a ratio to the absorbance of the untreated group.

### 2.9 Real-time quantitative polymerase chain reaction analysis

B16-F10 cells were cultured in 24-well plates (1.0 × 10^4^ cells/well). The cells were then treated with A-ELNs (5, 10, or 20 μg/mL) or the positive control, arbutin (1.0 mM) (Wako, Osaka, Japan), and simultaneously stimulated with 0.20 μM α-MSH, followed by incubation at 37 °C. RNA was extracted after 8 h for determining the expression of microphthalmia-associated transcription factor (*Mitf*), or after 48 h to determine the expression of tyrosinase (Tyr), tyrosinase-related protein 1 (Tyrp1), and DOPA chrome tautomerase (Dct). Total RNA was extracted from B16-F10 cells using the RNeasy Mini Kit (Qiagen, Hilden, Germany) and reverse-transcribed using the PrimeScript RT Reagent Kit (Takara Bio, Otsu, Japan). TaqMan quantitative polymerase chain reaction (qPCR) was performed using the StepOnePlus™ Real-Time PCR System (Thermo Fisher Scientific, Santa Clara, CA, USA). The mRNA expression levels of *Mitf*, *Tyrp1*, *Dct*, and *Tyr* were normalized to those of *Gapdh*. The PCR primers and TaqMan probes for *Gapdh* (Mm99999915_g1), *Mitf* (Mm00434954_m1), *Tyrp1* (Mm00453201_m1), *Dct* (Mm01225584_m1), *Tyr* (Mm00495818_m1), and TaqMan Universal PCR Master Mix were purchased from Applied Biosystems (Foster City, CA, USA).

### 2.10 Statistical analysis

All statistical analyses were performed using EZR version 1.29 (Saitama Medical Center, Jichi Medical University, Saitama, Japan). Data are expressed as means ± standard deviation. One-way analysis of variance (ANOVA) was performed to examine the significance of differences between treatments. Multiple comparison tests were performed using Tukey's test. Statistical significance was set at *P* < 0.05.

## 3 Results

### 3.1 **Characterization of *ELNs* obtained from** A. lancea rhizomes

**Fig. 1:**
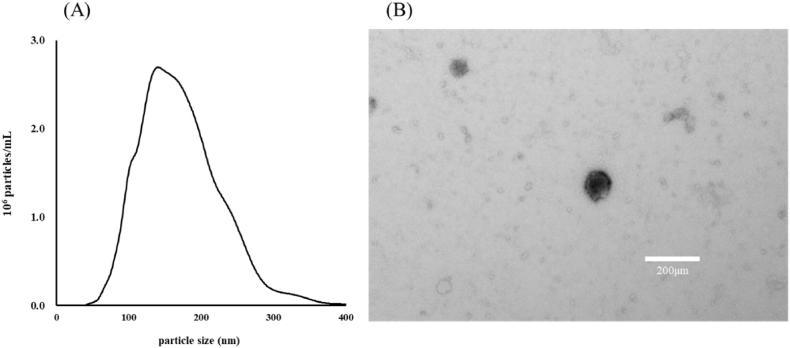
Characterization of A-ELNs (A) Size and yield of A-ELNs and (B) morphology of ELNs under TEM. A-ELNs: exosome-like nanoparticles from dried rhizome of *Atractylodes lancea*.

*A. lancea* rhizomes yielded 20.0 ± 4.4 mg ELNs. TEM analysis showed that these ELNs were round and their size ranged from 34 to 401 nm (Fig. 1A and B). Next, we compared the sequences of miRNAs found in A-ELNs with the miRNA library of *A. thaliana* and identified three miRNAs (ath-miR166f, ath-miR162a-5p, and ath-miR162b-5p) (Table 1)

**Table 1:** miRNAs identified in A-ELNs cargo after comparing the miRNA load of A-ELNs with the miRNA library of *Arabidopsis thaliana*.

miRNA name	Number of Reads
ath-miR166f	46
ath-miR162a-5p	2
ath-miR162b-5p	2

### 3.2 Effect of A-ELNs on B16-F10 cell viability

**Fig. 2:**
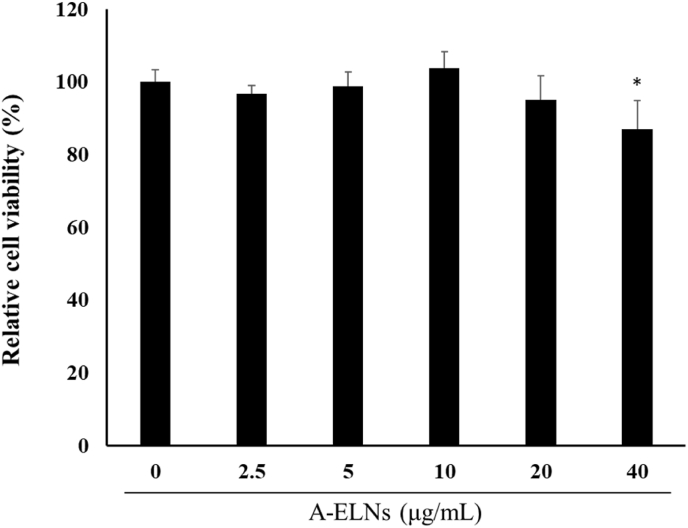
Effect of A-ELNs on B16-F10 cells viability, B16-F10 cell viability was measured 48 h after treatment with A-ELNs (0, 5, 10, 20, or 40 μg/mL). Data were evaluated using one-way ANOVA followed by Tukey's test. (mean ± SD, *P < 0.05 compared to the α-MSH-stimulated group). A-ELNs: exosome-like nanoparticles from dried rhizome of *Atractylodes lancea*.

We examined the effect of A-ELNs on the viability of B16-F10 cells. A-ELNs had no effect at 2.5–20 μg/mL but were significantly toxic at 40 μg/mL (*P* = 0.022) (Fig. 2), which was confirmed by ANOVA (*P* = 0.005).

### 3.3 *A-ELNs* uptake by *B16-F10* cells

**Fig. 3:**
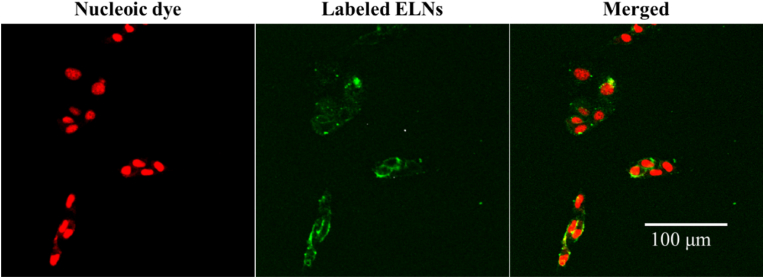
A-ELNs uptake by B16-F10 cells, B16-F10 cells were incubated with stained A-ELNs (20 μg/mL) for 3 h. The cells were observed using a confocal laser scanning microscope. A-ELNs: exosome-like nanoparticles from dried rhizome of *Atractylodes lancea*.

Labelled A-ELNs were incubated with B16-F10 cells. After 3 h incubation, A-ELNs were taken up by the cells, and no fluorescent signal was detected in the control (treated with only the same amount of staining reagent without A-ELNs) (Fig. 3).

### 3.4 Effect of *A-ELNs* on melanin content and intracellular tyrosinase activity

**Fig. 4:**
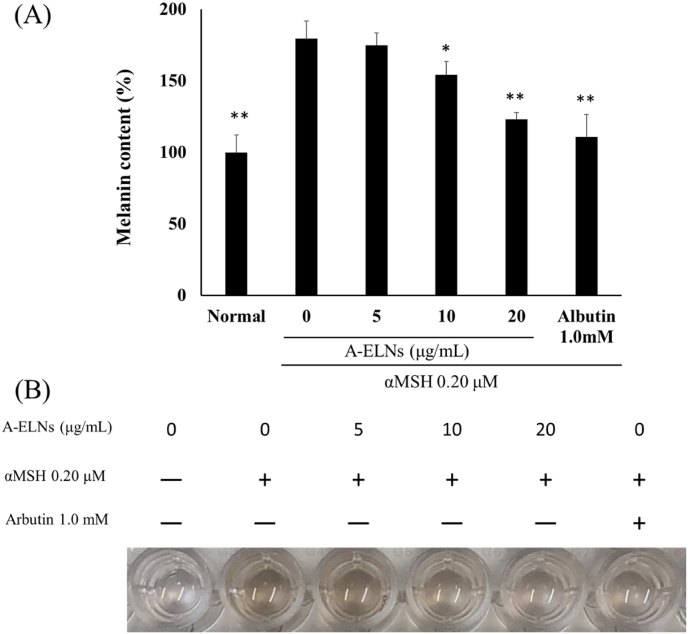
Effect of A-ELNs on melanin synthesis in B16-F10 cells. B16-F10 cells were treated with A-ELNs (5, 10, or 20 μg/mL) and 0.20 μM α-MSH for 72 h, and intracellular melanin content was measured at 360 nm. (A) Melanin contents relative to blank and positive control (1.0 mM arbutin). (B) Melanin accumulation in response to different treatments. The melanin content (%) of each sample was calculated as the ratio to the absorbance of the untreated group. Data were evaluated using one-way ANOVA followed by Tukey's test. (mean ± SD, *P < 0.05, **P < 0.01 compared to the α-MSH-stimulated group). A-ELNs: exosome-like nanoparticles from dried rhizome of *Atractylodes lancea*.

**Fig. 5:**
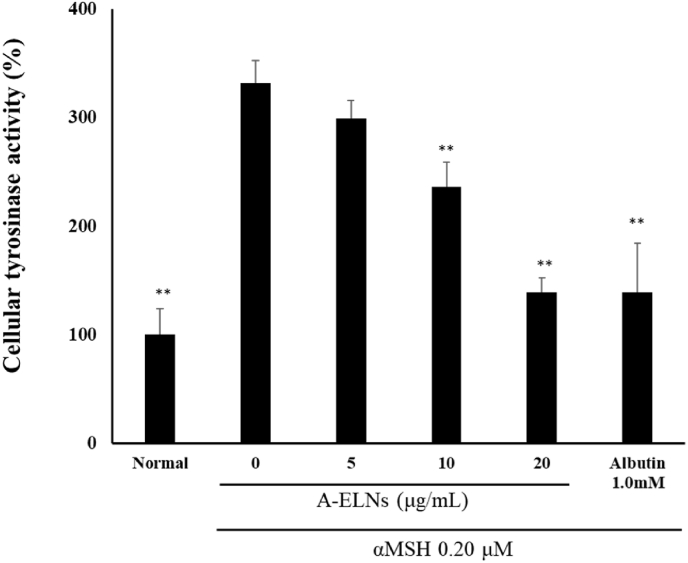
Effect of A-ELNs on intracellular tyrosinase activity. B16-F10 cells were treated with A-ELNs (5, 10, or 20 μg/mL) and 0.20 μM α-MSH for 72 h, and intracellular tyrosinase activity was measured. Tyrosinase activity (%) of each sample was calculated as a ratio to the absorbance of the untreated group. Data were evaluated using one-way ANOVA followed by Tukey's test. (mean ± SD, *P < 0.05, **P < 0.01 compared to the α-MSH-stimulated group). A-ELNs: exosome-like nanoparticles from dried rhizome of *Atractylodes lancea*.

We measured melanin content in B16-F10 cells treated with A-ELNs and α-MSH. Melanin content (*P* < 0.01) and intracellular tyrosinase activity (*P* < 0.01) were significantly increased by α-MSH stimulation; however, A-ELN treatment (10 and 20 μg/mL) significantly reduced these levels (Fig. 4A and B, and Fig. 5).

### 3.5 Effect of *ELNs* on melanogenesis-related gene expression

**Fig. 6:**
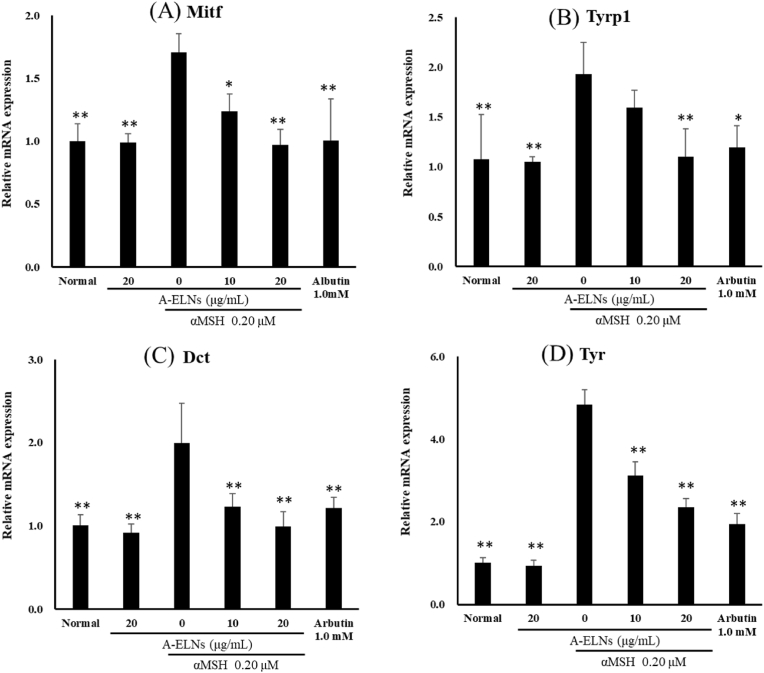
Effect of A-ELNs on the mRNA levels of melanogenesis-related factors in B16-F10 cells. B16-F10 cells were incubated with A-ELNs (5, 10, or 20 μg/mL) and 0.20 μM α-MSH. After 8 h, mRNA expression level of (A) *Mitf* was measured. After 48 h, the mRNA expression levels of (B) *Tyrp1*, (C) *Dct*, and (D) *Tyr* were measured. Data were evaluated using one-way ANOVA followed by Tukey's test. (mean ± SD, *P < 0.05, **P < 0.01 compared to the α-MSH-stimulated group). A-ELNs: exosome-like nanoparticles from dried rhizome of *Atractylodes lancea*.

We examined the effects of A-ELNs on the mRNA expression of melanogenesis-related factors. Although α-MSH stimulation increased the mRNA expression of the factors, A-ELNs significantly reduced the levels of *Mitf* (*P* < 0.01), *Tyrp1* (*P* < 0.01), *Dct* (*P* < 0.01), and *Tyr* (*P* < 0.01) (Fig. 6 A–D). These factors were not affected by A-ELN treatment in α-MSH-unstimulated B16-F10 cells.

## 4 Discussion

This is the first study to isolate and characterize ELNs from a hot water extract of *Atractylodes lancea* rhizomes. These A-ELNs contained three mature miRNAs and inhibited melanin production in α-MSH-stimulated B16-F10 cells. Thus, A-ELNs may have potential as a new whitening agent in pharmaceuticals and cosmetics.

Extracellular vesicles (EVs) are responsible for signalling among cells as they carry a cargo of functional biomolecules that regulate various physiological functions [18,19]. In cosmetic and pharmaceutical sciences, research studies have focused on developing melanin synthesis inhibitors using mammalian cell-derived EVs. Among them, human amniotic stem cell derived EVs were shown to inhibit melanin synthesis [20]. However, mammalian cell-derived EVs may also carry pathogens, potentially making their application in pharmaceuticals difficult [21]. Thus, plant cell-derived ELNs are a safer alternative for use in health research [22,23]. Although these ELNs primarily regulate plant physiology, some ELNs also evoke responses in animals. In mouse models, grape-derived ELNs protects against dextran sulfate sodium-induced colitis [24], and shiitake mushroom-derived ELNs protect the liver from acute injury [25]. Thus, plant derived ELNs have therapeutic potential for human diseases. Earlier, ELNs derived from the leaves of *Dendropanax morbifera* were shown to exert inhibitory effects on melanin synthesis [26]. However, this research area is nascent but ripe for further research. Here, we evaluated whether ELNs can inhibit melanin overproduction and be useful in the cosmetic and pharmaceutical field.

In this study, we examined the inhibitory effect of ELNs isolated from the rhizome of *A. lancea* on melanin production. This plant is a perennial herb that is widely distributed in East Asia, and it has long been used to treat digestive disorders and is also a well-known crude drug used in traditional Japanese Kampo medicines [[27], [28], [29]]. Although most plant derived ELNs have been isolated from fresh products [12], they can also be extracted from dried products [30]. Because *A. lancea* rhizome is used as a crude drug in Kampo medicine, it is readily available in dried form. Moreover, we used dried rather than fresh rhizomes because their large quantities may be needed in the future.

Three mature miRNAs were identified in A-ELNs. Among these, ath-miR166f was highly expressed. The miR166 family plays an important role in plant responses to abiotic stress [31]. For example, miR166 expression is induced by hypertonicity- and infection-related stress, and regulates the genes required to maintain homeostasis in response to these threats [32,33]. In addition, miR166g has been identified in ELNs derived from strawberries and reportedly reduces oxidative stress in human mesenchymal stromal cells [34]. The mechanism by which the miR166 family affects melanin synthesis remains unknown, but these miRNAs are believed to be the active components of A-ELNs. In addition to miRNAs, plant derived ELNs are reported to contain vitamins and proteins [35,36], which may contribute to their whitening effects. Therefore, additional studies are necessary to identify and characterize the active components of A-ELNs.

Plant-derived ELNs have different cellular destinations depending on their membrane structure and lipid content [37]. In this study, A-ELNs were taken up by B16-F10 cells where they exerted inhibitory effects on melanin synthesis. Although activity of melanin-synthesis related enzymes was affected by A-ELNs in our study, future studies should focus on identifying other intracellular targets of A-ENLs.

B16-F10 cells overproduce melanin when stimulated with UV-B or α-MSH, and several whitening components have been evaluated using these cells as an experimental model [38], including A-ENLs in our study. We found that A-ELNs suppressed melanin production in B16-F10 cells stimulated with α-MSH. Tyrosinase plays a vital role in melanin synthesis [39] and its expression can be inhibited by components isolated from natural products [38]. Moreover, the transcription factor, *Mitf,* regulates tyrosinase production by binding to the promoter regions of melanogenic enzymes [40,41]. In this study, A-ELNs decreased the mRNA expression of tyrosinases, such as *Tyrp1*, *Dct*, and *Tyr*, and reduced tyrosinase activity in B16-F10 cells. These results suggest that A-ELN suppresses melanogenic enzymes expression by downregulating *Mitf*, thereby resulting in reduced melanin synthesis.

Despite presenting some interesting findings, our study has some limitations. First, our conclusions are based on in vitro experiments, and should be validated in *in vivo* and preclinical models. As cosmetic agents, A-ELNs would be topically applied. However, melanocytes present in the epidermal basal layer synthesize melanin, and future formulations will need to ensure that these A-ENLs can migrate and act on the basal epidermis regardless of the innate effects of their phospholipid bilayer [37]. Plant-derived ELNs have been reported to have the ability to pass through biological membranes [43], which may allow them to penetrate the skin more easily and be more effective than conventional drugs. Second, the active components within A-ELNs need to be identified. Although we comprehensively analysed the miRNA in A-ELNs, vitamins and proteins present in ELN cargo may have contributed to the melanin-suppressing effects. Third, we extracted A-ELNs from dried rhizome using hot water in this study, but the extraction efficiency, composition, and number of active components in A-ELNs may change if fresh rhizomes are used or ELNs are extracted at different water temperatures. Thus, the method for extracting A-ELNs from *A. lancea* rhizomes needs to be optimized, which may also contribute to the quality of A-ELNs. However, drying plants is often advantageous in terms of long-term preservation and transportation. The finding in this study that ELNs could be extracted from dried plants may be very useful.

Although several melanogenesis inhibitors have been developed, many of these exert undesirable side effects, such as erythema, itching, and leukoderma; therefore, new treatment strategies need to be devised to treat these patients [8,9,42]. Results in this study suggest A-ELN suppresses melanogenic enzymes expression by downregulating *Mitf*, resulting in reduced melanin synthesis. Therefore, A-ELN has potential as a new whitening agent in pharmaceuticals and cosmetics. This is the first report to investigate that plant-derived ELNs inhibit melanin production. We expect this to be a landmark report that will facilitate research on whitening agents.

## Author contributions

Participated in research design: Ishida, Jobu, Miyamura.

Conducted experiments: Ishida, Morisawa, Kawada, Yoshioka.

Performed data analysis: Ishida, Kawada.

Wrote or contributed to the writing of the manuscript: Ishida, Morisawa, Jobu, Kawada, Miyamura.

## Declaration of competing interest

The authors declare that they have no known competing financial interests or personal relationships that could have appeared to influence the work reported in this paper.

## Data Availability

Data will be made available on request.
